# The Role of MRI and CT Scan in Classification and Management of Pelvic Fractures: A Systematic Review

**DOI:** 10.7759/cureus.52215

**Published:** 2024-01-13

**Authors:** Dalal H Almansouri, Ayat I Elsherbini, Manal Alharthi, Shatha ALotaibi, Lamyaa Alshehri

**Affiliations:** 1 Radiologic Technology, Specialized Dental Center, Taif, SAU; 2 Radiology, Faculty of Medicine, Tanta Univerisity, Tanta, EGY; 3 Radiology Technology, Alwadi Primary Health Care, Riyadh, SAU; 4 Radiological Technology, King Abdulaziz Specialist Hospital, Taif , SAU; 5 Radiologic Technology, Primary Health Care, King Abdulaziz Medical City for National Gurad, Jeddah, SAU

**Keywords:** systematic review, management, ct, mri, pelvic fractures

## Abstract

The mortality risk for individuals with pelvic fractures ranges from 10% to 50%, depending on the severity of the bleeding and the presence of concurrent brain, thorax, and abdomen injuries. This systematic review aims to comprehensively investigate the role of MRI and CT in diagnosing and managing pelvic fractures. PubMed, SCOPUS, Web of Science, and Science Direct were systematically searched for relevant literature. The keywords "Pelvic fractures," "Diagnosis," "Computed tomography," "CT," "Magnetic resonance imaging," and "MRI" were converted into PubMed Mesh terms and used to find the relevant studies. Rayyan Qatar Computing Research Institute (QCRI) was employed throughout this comprehensive process. The systematic review included publications with full English text, available free articles, and human trials among adults. This review included 12 studies with 1,798 patients, and 935 (52%) of them were females. Two articles were prospective, and 10 articles were retrospective. In conclusion, the diagnosis and management of pelvic fractures require a tailored approach based on patient characteristics, injury mechanisms, and hemodynamic status. Because MRI detects a high number of concealed sacral fractures, it provides greater sensitivity and diagnostic validity in identifying acute pelvic fractures. Moreover, MRI is effective in diagnosing occult pelvic fractures and detecting soft tissue anomalies. However, MRI is unlikely to replace CT as the gold standard in the initial diagnosis of pelvic fractures; CT may also be preferable to MRI due to shorter emergency department (ED) time and the significant proportion of elderly patients who are contraindicated to MRI. Additionally, CT scanning aids in determining the need for emergent angiographic embolization and facilitates surgical planning.

## Introduction and background

Pelvic fractures are significant injuries that can lead to a variety of complications. They accounts for approximately 1.5%-3% of all bone injuries [1]. The mortality rate for patients suffering from pelvic fractures ranges from 10% to 50%, depending on the severity of the bleeding and the occurrence of accompanying injuries to the brain, thorax, and abdomen [2-4]. The majority of pelvic fractures are caused by high-energy trauma. Other reasons include falls from great heights, heavy object falls, and so on [5]. Patients with pelvic injuries frequently have multiple systemic ailments, which increase morbidity and mortality.

Although pelvic fractures are uncommon, injuries to other locations are common as a result of the energy necessary to create a pelvic fracture [6]. The thorax, long bones, brain, abdominal organs, and spine are the most commonly implicated structures. Making an early decision to explore the pleural space or peritoneum when there is suspicion of bleeding or intestinal injury can significantly reduce the risk of death or serious complications. In most cases, this decision can be made during the primary survey. It is important to note that more extensive diagnostic procedures can be deferred until bleeding is under control. Once ongoing bleeding has been controlled or ruled out based on clinical assessment, the secondary survey should prioritize identifying any pelvic injuries that could contribute to blood loss or complications [7].

Plain radiography (anterior-posterior (AP), inlet, and exit X-rays) and thin-cut (3-mm) CT scans are required to classify pelvic fractures and dislocations. If possible, the AP pelvis film is taken to prevent blurring landmarks before bladder catheterization and cystography. The classification of pelvic fractures serves two purposes: it provides a standard language for communication among medical professionals caring for the patient and a means of linking clinical outcomes to prognosis [7]. Nevertheless, plain radiography can predict pelvic fracture, in up to 10% of patients, and some fractures can be missed [8]. Accordingly, this systematic review aims to comprehensively investigate the role of MRI and CT in diagnosing and managing pelvic fractures.

## Review

Methodology

This systematic review complied with established criteria (Preferred Reporting Items for Systematic Reviews and Meta-Analyses, PRISMA) [9].

Study design and duration

This descriptive systematic review was conducted in September 2023. The results were summarized while no meta-analysis was performed.

Search strategy

A thorough search of four major databases, including PubMed, SCOPUS, Web of Science, and Science Direct, was done to find the relevant literature. We restricted our search to English and considered each database's unique requirements. The following keywords were converted into PubMed Mesh terms and used to find the relevant studies: "Pelvic fractures," "Diagnosis," "Computed tomography," "CT," "Magnetic resonance imaging," and "MRI." The Boolean operators "OR" and "AND" matched the required keywords. Publications with full English text, available free articles, and human trials were among the search results.

Selection Criteria

We considered the following criteria for inclusion in this review:

· Study designs that investigated the diagnosis of pelvic fractures using CT and/ or MRI

· Only adults were included (>18 years)

· Only human subjects

· English language

· Free accessible articles

Data Extraction

Rayyan Qatar Computing Research Institute (QCRI) was used to check the output of the search technique for duplication [10]. The researchers assessed the titles' and abstract relevance by altering the combined search results with a set of inclusion/exclusion criteria. The reviewers thoroughly scrutinized each paper that matched the inclusion criteria. The writers discussed dispute-resolution approaches. The authorized study was uploaded using a previously generated data extraction form. The authors extracted data about the study titles, authors, study year, country, participants, gender, diagnostic method, and primary outcomes. A separate sheet was created for the risk of bias assessment.

Strategy for Data Synthesis

Summary tables were created using data from relevant studies to provide a qualitative interpretation of the findings and study components. We analyzed and summarized the findings from the selected studies thematically according to the inclusion criteria. 

Risk of Bias Assessment

The quality of the included studies was assessed using the Risk of Bias in Non-randomised Studies of Interventions-I (ROBINS-I) assessment approach for non-randomized trials of treatments [11]. Confounding, participant selection for the study, classification of interventions, deviations from intended interventions, missing data, assessment of outcomes, and selection of the reported result were the seven themes evaluated.

Results

Search Results

**Figure 1:**
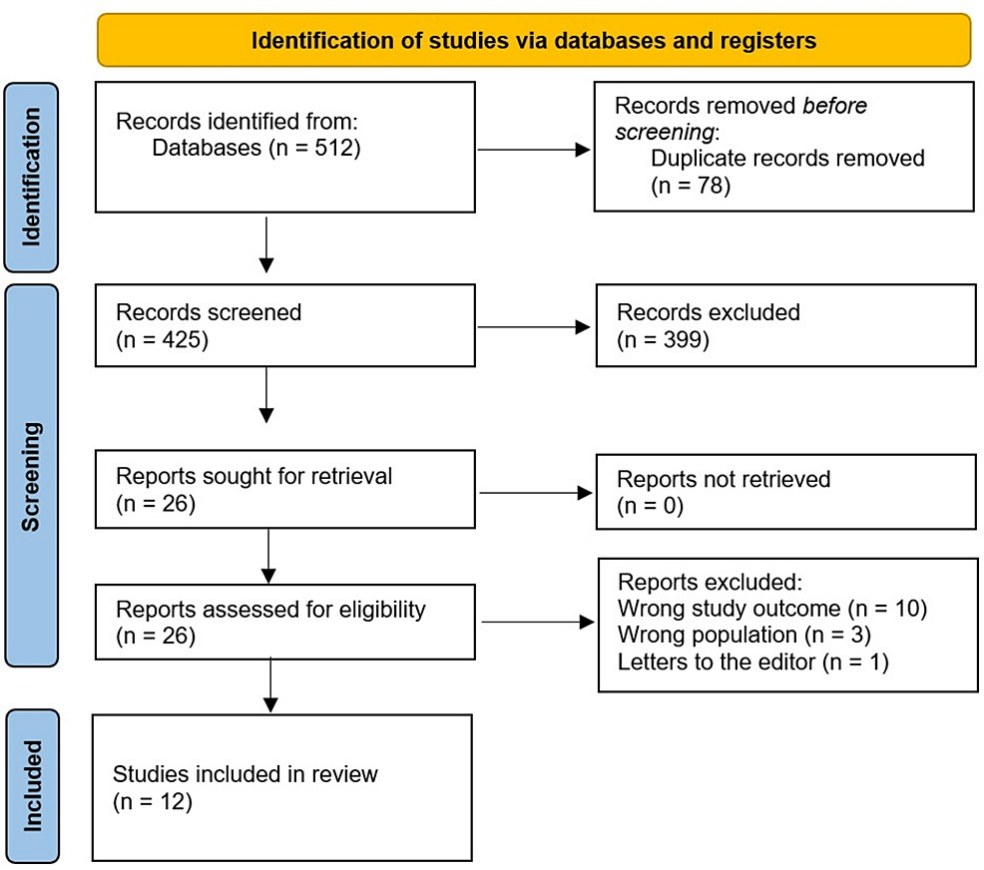
PRISMA flowchart summarizes the study selection process.

A total of 512 study articles resulted from the systematic search, and 87 duplicates were deleted. Title and abstract screening were conducted on 425 studies, and 399 were excluded. 26 reports were sought for retrieval, and no articles were retrieved. Finally, 26 studies were screened for full-text assessment; 10 were excluded for irrelevant study outcomes, three for the irrelevant population type, and one article was a letter to the editors. 12 eligible study articles were included in this systematic review. A summary of the study selection process is presented in Figure 1.

Characteristics of the Included Studies

Table 1 presents the sociodemographic characteristics of the included study articles. Our results included 12 studies with 1,798 patients, and 935 (52%) were females. Two articles were prospective [12,13]. 10 articles were retrospective [14-23]. 

**Table 1:** Sociodemographic characteristics of the included participants. NM: Not mentioned

Study	Study design	Country	Participants	Mean age (years)	Males (%)
Henes et al., 2012 [12]	Prospective	Germany	38	74.7	31 (81.6)
Cabarrus, et al., 2008 [14]	Retrospective	USA	145	67.7 ± 16.2	104 (71.1)
Gandhi et al., 2018 [18]	Retrospective	India	6	NM	3 (50)
Palm et al., 2020 [22]	Retrospective	Germany	46	NM	40 (86.9)
Pereira et al., 2000 [17]	Retrospective	USA	290	39 ± 1.1	122 (42.1)
Fu et al., 2009 [23]	Retrospective	Taiwan	108	41.1 ± 20.9	54 (50)
Lang et al., 2020 [15]	Retrospective	Germany	140	62	75 (53.6)
Ghisla et al., 2018 [19]	Retrospective	Switzerland	21	57.8 ± 18.7	7 (33.3)
Nüchtern et al., 2015 [13]	Prospective	Germany	60	74.7 ± 15.6	53 (88.3)
Chan et al., 2023 [20]	Retrospective	Taiwan	444	43.2 ± 18.3	177 (39.9)
Benjamin et al., 2022 [21]	Retrospective	USA	285	46	117 (41.1)
Eggenberger et al., 2019 [16]	Retrospective	USA	215	76	153 (71.2)

Table 2 presents the diagnostic method used and the outcomes of the included studies. Five studies compared MRI to CT in pelvic fracture diagnosis [12-16]. MRI has superior sensitivity and diagnostic validity in diagnosing acute pelvic fractures [12-14]. One study concluded that MRI is unlikely to become the gold standard in the initial diagnosis of pelvic fractures [13]. The use of dynamic helical CT scanning early in the treatment of a multiply injured patient with a pelvic fracture precisely determines the requirement for emergent angiographic embolization [17].

**Table 2:** Clinical characteristics and outcomes of the included studies ROBIN: ; DECT: Dual-energy CT; ED: Emergency department; FFP: Fresh frozen plasma; MDCT: Multidetector CT; PXR: Pelvis x-ray; SI: Sacro-iliac

Study	Treatment/ diagnostic method	Main outcomes	ROBIN-I
Henes et al., 2012 [12]	CT/MRI	When compared to routinely performed MDCT, MRI has superior sensitivity and diagnostic validity in the diagnosis of acute pelvic fractures. Because MRI detected a high number of concealed sacral fractures, they believe that MRI is appropriate for patients with a suspicion of a posterior pelvic ring fracture but negative CT findings following trauma.	Moderate
Cabarrus, et al., 2008 [14]	CT/MRI	In diagnosing insufficiency fractures of the pelvis, MRI performed significantly better than CT.	Moderate
Gandhi et al., 2018 [18]	CT	CT-guided SI joint stabilization has several advantages, including safer and more accurate screw placement, shorter operating times, less blood loss, early final fixation, rapid mobilization, and fewer infections and wound problems.	High
Palm et al., 2020 [22]	DECT	In terms of edema identification and specific fracture grading in FFP, DECT was found to be trustworthy and superior to conventional CT. DECT thus combines the benefits of traditional CT (excellent visibility of bone substance) with MRI (visualization of the medullary cavity and occult fractures).	Moderate
Pereira et al., 2000 [17]	CT	The use of dynamic helical CT scanning early in the treatment of a multiply injured patient with a pelvic fracture precisely determines the requirement for emergent angiographic embolization.	High
Fu et al., 2009 [23]	CT/PXR	Although a CT scan is more sensitive in detecting acetabular or minor pelvic fractures, PXR is adequate for assessing pelvic fracture stability early. Early angioembolization is recommended for patients with an unstable pelvic fracture, according to the current series.	High
Lang et al., 2020 [15]	MRI/CT/X-ray	In the case of solitary pelvic ring fractures, MRI was conducted, particularly in older female patients with pelvic fractures with inadequate trauma. Instead, isolated CT diagnoses are concerned with the young guy following high-energy trauma.	Moderate
Ghisla et al., 2018 [19]	3D-CT	This study recorded the treatment of a significant number of patients with pelvic ring fractures using intraoperative 3D-CT O-Arm assisted navigation. This method allowed for precise and secure SI screw alignment. When compared to published statistics for conventional procedures, the rate of misplacement was reduced, and no difficulties arose.	Moderate
Nüchtern et al., 2015 [13]	CT/MRI	A CT scan is still the gold standard for detecting displaced pelvic fractures in individuals with fractures in the anterior pelvis. Because of its scarcity, MRI is unlikely to become the gold standard in the initial diagnosis of pelvic fractures.	Low
Chan et al., 2023 [20]	3D-CT	A single-stage CT scan considerably reduced the time to definitive pelvic fixation, was utilized to assess the presence or absence of internal organ injury or haemorrhage, and was integrated with 3D pictures to aid surgical planning. Patients could have early surgical fixing without exposing themselves to unneeded radiation or travelling.	Low
Benjamin et al., 2022 [21]	CT/X-ray	In the emergency room, a pelvic X-ray may be helpful in diagnosing severe pubic symphysis diastasis. It does, however, miss or underestimate a considerable number of fractures. Patients with a suspected cause or clinical suspicion of pelvic fracture should have a CT scan evaluated.	Moderate
Eggenberger et al., 2019 [16]	CT/MRI	Although MRI has a higher sensitivity for fractures, our investigation demonstrated that CT was sufficient to rule out hip and pelvic fractures in this patient population. CT may also be preferable to MRI due to reduced ED time and the high proportion of elderly patients who are contraindicated to MRI.	Moderate

A single-stage CT scan considerably reduced the time to definitive pelvic fixation, was utilized to assess the presence or absence of internal organ injury or hemorrhage, and was integrated with 3D pictures to aid surgical planning [18-20]. In the emergency room, a pelvic X-ray may help diagnose severe pubic symphysis diastasis. It does, however, miss or underestimate many fractures [21]. CT may also be preferable to MRI due to reduced ED time and the high proportion of elderly patients contraindicated to MRI [16].

Discussion

The diagnostic and therapeutic techniques used to treat pelvic ring fractures are determined by the patient's features, the mechanism of injury, and the patient's hemodynamic status at the time of presentation. Understanding the complicated anatomy and biomechanics of pelvic stability may help to determine proper initial therapy strategies [24]. This systematic review aims to comprehensively investigate the role of MRI and CT in diagnosing and managing pelvic fractures.

Regarding the role of MRI in the management of pelvic fracture, in prior small reports, MRI is a sensitive imaging method that is effective in detecting and characterizing occult traumatic bone injuries [25,26]. Some experts consider it to be the investigation of choice in patients with clinical suspicion of a femoral fracture but normal radiographs [27]. In the current review, five studies compared MRI to CT [12-16]. The findings showed that MRI is superior as an imaging technique compared to CT in acute pelvic fractures [12-14]. In particular, MRI has the advantage of detecting the sacrum, femoral head, and acetabulum or if reduced bone density is suspected.

However, there is controversy in the role of MRI as a golden standard. Some studies suggested that MRI should be the primary diagnostic technique when insufficiency or occult pelvic fractures are suspected or to be an established technique where negative radiographic findings are present [12,14]. However, Nüchtern et al. reported that MRI is unlikely to become the gold standard in the initial diagnosis of pelvic fractures [13]. Besides, the elderly population may have implants or medical devices that prevent them from using MRIs. In the present review, two of the included studies assessed MRI in comparison to CT among the elderly [15,16]. The results suggest that MRI has no added value to CT in diagnosing fractures in this population. Additionally, the length of stay in ED was lesser for patients who underwent CT. While the fractures diagnosed by MRI may not differ in the management.

CT is not typically used to detect occult fractures unless there is a specific contraindication to MRI, and cases where this was the case were not included in our investigation. The procedure described above is prevalent in many centers and follows national norms [27]. Concerning the role of CT in detecting pelvic fracture in the present systematic review, five studies analyzed the efficiency of CT only [17-20,22]. The early using of dynamic helical CT aided in identifying the requirement of emergent angiographic embolization in patients with traumatic pelvic fractures [17]. A pelvic ring fracture is a high-energy injury that should be considered in any patient with a suspicious mechanism (car accident, crush injury, or fall from a height). The stability of the pelvic ring, which may be examined clinically and radiographically, should emphasize the diagnosis of individuals with a pelvic ring fracture [24]. For patients treated with pelvic ring fracture, intraoperative 3D CT scan accurately allowed safe screw fixation [19,20]. This strategy showed improvement in patient outcomes by providing timely surgical interventions and reducing delays due to the need for additional CT scans.

Furthermore, two studies compared CT scans to X-rays [21,23]. A CT scan could more accurately identify acetabular or small pelvic fractures than an X-ray. On the other side, X-rays compared to CT, underestimates a substantial amount of fractures. In a previous study, pelvic X-ray had some limitations including limited sensitivity in detecting certain types of fractures, such as posterior pelvic ring fractures [28]. In addition, the interpretation of pelvic X-rays relies on the experience and expertise of the radiologist or clinician, which can introduce variability and potential diagnostic errors [29]. Historically, radiographic outcome metrics have been the most commonly reported in orthopedic trauma. These are notoriously difficult to standardize, and digital X-rays have made things even more complicated through the variability in image acquisition and interpretation [30,31]. Expert opinion and historical records of non-operatively treated fractures indicate that large displacement of pelvic ring disturbances results in poor outcomes; however, the measurement technique for quantifying displacement has yet to be developed [30,32].

## Conclusions

MRI has superior sensitivity than CT in diagnosing acute pelvic fractures. It has an advantage in detecting the sacrum, femoral head, and acetabulum or when there is reduced bone density. CT is preferable to MRI due to reduced ED time and the high proportion of elderly patients who are contraindicated to MRI. 3D-CT imaging is a valuable tool to improve surgical planning in patients treated with pelvic ring fractures. A PXR can miss many fractures in the ED.
